# Modeling the Regulatory Mechanisms by Which NLRX1 Modulates Innate Immune Responses to *Helicobacter pylori* Infection

**DOI:** 10.1371/journal.pone.0137839

**Published:** 2015-09-14

**Authors:** Casandra W. Philipson, Josep Bassaganya-Riera, Monica Viladomiu, Barbara Kronsteiner, Vida Abedi, Stefan Hoops, Pawel Michalak, Lin Kang, Stephen E. Girardin, Raquel Hontecillas

**Affiliations:** 1 Center for Modeling Immunity to Enteric Pathogens, Virginia Bioinformatics Institute at Virginia Tech, Blacksburg, VA, United States of America; 2 Nutritional Immunology and Molecular Medicine Laboratory, Virginia Bioinformatics Institute at Virginia Tech, Blacksburg, VA, United States of America; 3 Virginia-Maryland Regional College of Veterinary Medicine, Blacksburg, VA, United States of America; 4 Laboratory of Medicine & Pathobiology, University of Toronto, Toronto, Ontario, Canada; Indian Institute of Science, INDIA

## Abstract

*Helicobacter pylori* colonizes half of the world’s population as the dominant member of the gastric microbiota resulting in a lifelong chronic infection. Host responses toward the bacterium can result in asymptomatic, pathogenic or even favorable health outcomes; however, mechanisms underlying the dual role of *H*. *pylori* as a commensal versus pathogenic organism are not well characterized. Recent evidence suggests mononuclear phagocytes are largely involved in shaping dominant immunity during infection mediating the balance between host tolerance and succumbing to overt disease. We combined computational modeling, bioinformatics and experimental validation in order to investigate interactions between macrophages and intracellular *H*. *pylori*. Global transcriptomic analysis on bone marrow-derived macrophages (BMDM) in a gentamycin protection assay at six time points unveiled the presence of three sequential host response waves: an early transient regulatory gene module followed by sustained and late effector responses. Kinetic behaviors of pattern recognition receptors (PRRs) are linked to differential expression of spatiotemporal response waves and function to induce effector immunity through extracellular and intracellular detection of *H*. *pylori*. We report that bacterial interaction with the host intracellular environment caused significant suppression of regulatory NLRC3 and NLRX1 in a pattern inverse to early regulatory responses. To further delineate complex immune responses and pathway crosstalk between effector and regulatory PRRs, we built a computational model calibrated using time-series RNAseq data. Our validated computational hypotheses are that: 1) NLRX1 expression regulates bacterial burden in macrophages; and 2) early host response cytokines down-regulate NLRX1 expression through a negative feedback circuit. This paper applies modeling approaches to characterize the regulatory role of NLRX1 in mechanisms of host tolerance employed by macrophages to respond to and/or to co-exist with intracellular *H*. *pylori*.

## Introduction

*Helicobacter pylori* is a microaerophilic Gram-negative, spiral-shaped bacterium that colonizes 50% of the world’s population establishing a decades-long infection as the dominant member of the human gastric microbiota [1]. *H*. *pylori* colonization results in health outcomes ranging from protection against allergies, diabetes, asthma and chronic inflammatory autoimmune diseases to favoring peptic ulcers and cancer. A small proportion (10–15%) of *H*. *pylori*-infected individuals experience serious pathologies [2]. Therefore investigating the complex tolerance mechanisms that facilitate the co-existence between *H*. *pylori* and its human host may yield a deeper understanding of mechanisms of immunoregulation at mucosal sites. Furthermore, characterizing innate immune mechanisms that reduce the negative impact of *H*. *pylori* infection on host fitness without directly affecting microbial burden may result in host-based precision medicine strategies that do not rely on anti-microbial treatment.

Mucosal pattern-recognition receptors (PRRs) expressed by epithelial cells and mononuclear phagocytes (MNP; macrophages and dendritic cells) are essential for detection of *H*. *pylori* and required for initiation of both effector and regulatory immune responses following infection [3]. The loss of a single PRR can cause dramatic changes in immunological parameters and disease outcomes. For example, loss of TLR2 signaling during *H*. *pylori* infection results in dysregulated host responses and severe immunopathology [4]. Conversely, NOD1-deficieny is associated with significantly reduced interferon production at the expense of higher *H*. *pylori* burden in the gastric mucosa [5]. Thus, characterizing the mechanisms underlying the modulation of PRR expression and signaling by *H*. *pylori* infection at the systems level may lend valuable new insights into the regulation of inflammatory and anti-inflammatory host responses, bacterial burden and tissue damage at the gut mucosa. Additionally, modulating host immunity through genetic manipulation of PRRs represents an effective means to better understand the mechanisms by which *H*. *pylori* endures as a commensal or pathogenic organism.

Although traditionally recognized mainly as an extracellular microbe, more recent evidence suggests that *H*. *pylori* can thrive as a facultative intracellular bacterium and replicate within gastric epithelial cells and mononuclear phagocytes (reviewed [6]). In our novel pig model of *H*. *pylori* infection we demonstrated presence of the bacterium inside myeloid rich lymphoid aggregates as well as the induction of cytotoxic CD8+ T cell responses by *H*. *pylori* infection [6, 7]. Additionally, we recently published a tissue-level computational and mathematical model that explores the role of macrophage phenotypes on disease pathogenesis [8–10]. In a follow up study, we established an *in vitro* co-culture system to study intracellular *H*. *pylori* and validated the importance of myeloid cells in maintaining bacterial loads by demonstrating reduced colonization following macrophage depletion *in vivo*. Although acknowledged, mechanisms underlying host responses to intracellular *H*. *pylori* are widely unresolved.

This study was designed to investigate immunomodulatory mechanisms of macrophages infected with *H*. *pylori*. Using global transcriptome sequencing we identify three temporal molecular modules (early, sustained and late) significantly altered in *H*. *pylori* infected bone marrow derived macrophages (BMDM) from mice. In addition, we characterize a cohort of genes significantly down-regulated during co-culture. Analysis of PRR expression in co-culture studies confirms that *H*. *pylori* infection suppresses regulatory nucleotide-binding domain and leucine-rich repeat-containing protein X1 (NLRX1). Many canonical PRR and cytokine signaling pathways modulated by *H*. *pylori* converge at NLRX1. Limited mechanistic insight exists for cell-specific immunomodulatory roles of NLRX1 as well as host-mediated or pathogen-driven suppression of this regulatory NLR. We present a validated computational model for characterizing novel feedback loop tolerance mechanisms implicated in the regulation of microbial burden and innate immune responses in macrophages.

## Results

### *H*. *pylori* induces distinct temporal waves in innate immunity in macrophages

**Fig 1:**
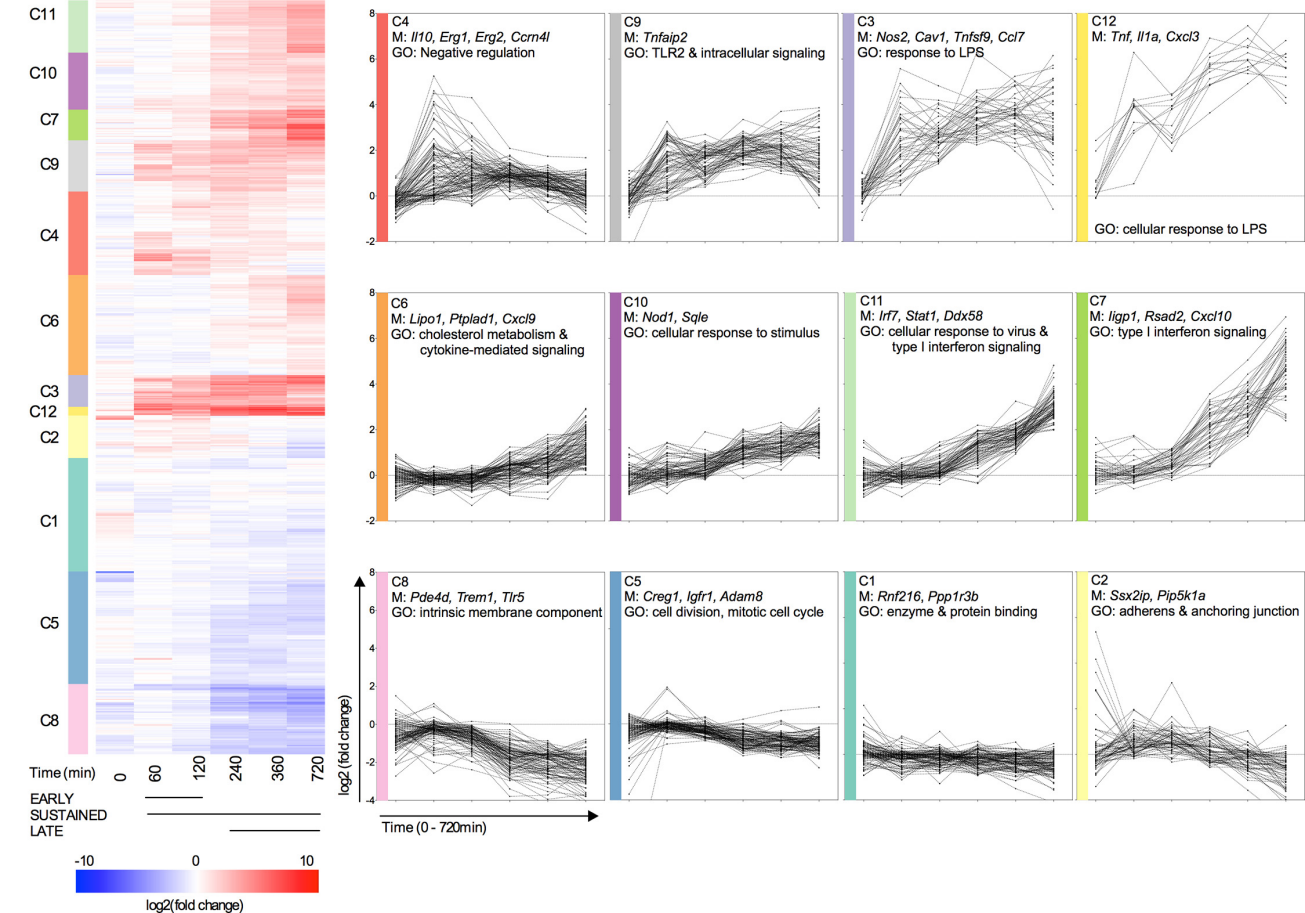
Intracellular *Helicobacter pylori* induces temporal waves in macrophage immunity. Clustering analysis was performed on differentially expressed genes (FDR p-value <0.05) obtained from RNAseq performed on macrophages co-cultured with *H*. *pylori*. Values are presented as the log2 (ratio) of average infected (n = 3) to average non-infected (n = 3) for each time point (0, 60, 120, 240, 360 and 720 min post-co-culture). Transcripts were grouped into 12 gene-modules based on expression levels and temporal behavior. Gene expression profiles for each cluster are plotted with representative members (M) and similarities in function identified by Gene Ontology (GO).

To study macrophage-specific responses to intracellular replication we co-cultured primary wild type (WT) bone marrow derived macrophages (BMDM) with *H*. *pylori* using a gentamycin protection assay and examined global gene expression at six discrete time points (0, 60, 120, 240, 360 and 720 minutes post-co-culture). Our analysis identified 1,077 differentially expressed genes due to infection in a time-dependent manner (FDR p-value < 0.05). Clustering was used to categorize significantly altered transcripts into 12 gene modules (Clusters 1–12). Within each cluster, genes have similar temporal magnitudes, synchronized expression patterns and common biological roles when analyzed by Gene Ontology (GO) enrichment [11, 12]. Chronologically, the profiles of the clusters broadly capture early transient (C4), sustained (C3, C9, C12), late (C6, C7, C10 C11) and suppressed (C8) patterns (**Fig 1**).

Genes with an early transient peak (C4) fall in a sub-cluster embodying regulatory molecules that negatively influence cellular stress and inflammation. *H*. *pylori* infection is known to induce a regulatory response in dendritic cells (DCs) characterized by enhanced production of IL-10. This dataset provides evidence that *H*. *pylori* infected macrophages up-regulate *Il10* mRNA through similar means early during infection (C4). We have further characterized this regulatory signature by identifying increased *E2f5*, *Erg1* and *Csrnp1*, molecules with anti-tumor properties, and *Prdm1*, whose gene product BLIMP-1 is a negative regulator of interferon (IFN) beta signaling. *Ddit4* (also referred to as *Redd1*), a glucose-regulated repressor of Akt/mTORC1 signaling and promoter of autophagy, was also among the rapidly induced transcripts [13–15]. Although dominated by immune suppressors, the early peak also contained pro-inflammatory mediators *Csf2* and *IL23a*.

The sustained innate response wave includes genes whose transcriptional profile is induced around 60 minutes and remains expressed throughout the duration of the experiment. Functionally, this phase corresponds to effector immunity elicited by recognition of extracellular bacteria and inflammatory cytokines. TLR2-dependent signaling is significantly enriched (C9) and likely governs the sustained response in a manner similarly described for *H*. *pylori* infected DCs [16]. Proinflammatory TLR2-mediated cytokines *Tnf*, *IL1a*, *Saa3* (C12) as well as *Il1b*, *Il6*, *Nos2*, *Ccl22* and *Ccl7* (C3) were all induced early and remained at high levels throughout the duration of the co-culture. The transcript for IL-12, an immunomodulatory cytokine involved in orchestrating T cell responses, clustered in C3. Antimicrobial peptides (AMP) are an important innate defense mechanism for epithelial cells to kill *H*. *pylori* and are generally enhanced with pro-inflammatory cytokines [17]. Interestingly we found that AMP were largely unchanged in our macrophage dataset; *Ltf* and *Lyz* cluster in C2 and were slightly down-regulated and neither β-defensins nor S100 genes were significantly altered. This suggests some degree of cell-specificity in response to intracellular versus extracellular *H*. *pylori* stimulation as well as differences in induction of host response between macrophages and epithelial cells.

The late wave contains genes with simultaneous onset of expression beginning at 240 minutes that continues to rise through the last collection. Late clusters are representative of host response to intracellular bacterial stimuli resulting in type I interferon signaling (C6-7, C10-11). *Nod1* (C10) and its responsive genes *Irf7* and *Stat1* (C11) were elevated late during infection as well as interferon-induced genes *Cxcl10*, *Ifit1-3*, *Iigp1* and *Rsad2* (C7). Similar to sustained genes, the later transcriptional pattern is not unique to macrophages and is observed in *H*. *pylori* treated DCs [16].

Down-regulated transcripts also coherently group together (C8) and signify genes that may be modulated by *H*. *pylori* to thrive in the host environment. Most C8 transcripts are intrinsic membrane associated molecules involved in signal transduction. Clusters 1,2 and 5 included genes associated, enzyme and protein binging, cell adhesion, and cell division respectively but were relatively unaltered. Moreover, these clusters contain transcripts with the highest p-values indicating that these three clusters may not be significant in this study.

Together these data suggest that mechanisms of mononuclear phagocyte immunity to *H*. *pylori* occur sequentially and may depend on the location of the bacterium with respect to the host cell. Additionally, *H*. *pylori* induces a unique regulatory gene module with an early transient peak during infection.

### Time-sensitive innate immune responses to *H*. *pylori* are induced by intracellular and extracellular PRRs in macrophages

**Fig 2:**
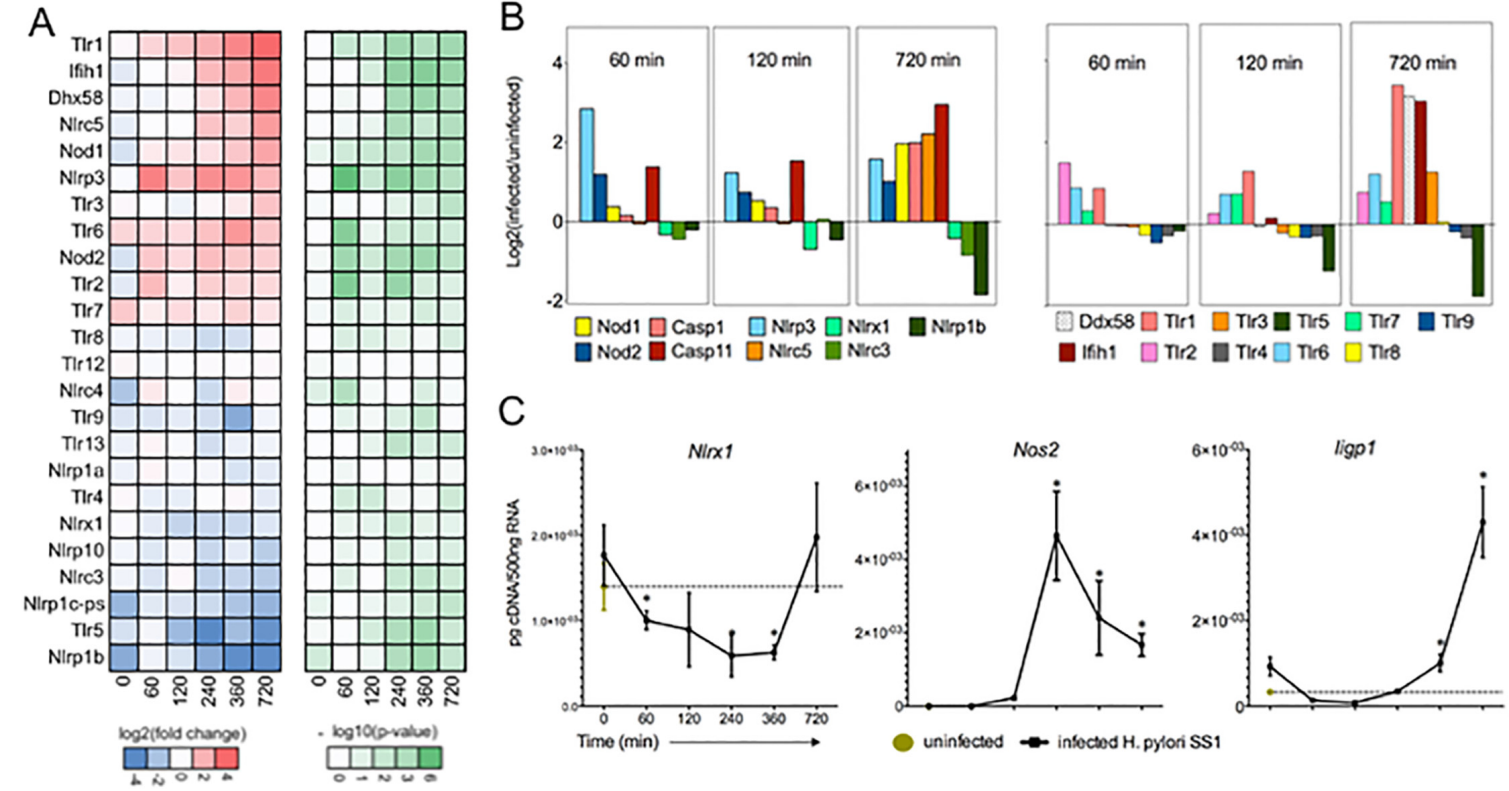
Pattern recognition receptors are differentially regulated by *Helicobacter pylori*. Genes involved in pathogen detection were extracted from the RNAseq dataset and analyzed at each time point for significance (A; left–expression, right–significance). Expression of effector PRRs conformed to sustained or late waves whereas regulatory molecules were suppressed (B). Dynamic expression of transcripts observed by RNAseq was validated by quantitative real time RT-PCR (C). Transcriptomic data (A,B) are presented as average log2(ratio) whereas validation gene expression is presented as pg cDNA/500ug RNA. Asterisk indicates *P*-value < 0.05 based on ANOVA.

Innate PRR families facilitate the recognition of unique components from microbes, host response and/or the environment to subsequently induce classic molecular cascades that efficiently modulate immunity. TLRs 2 and 9, RIG-I, NOD1, NOD2, NLRP3, NLRC4, NLRC5, NLRP9, NLRP12 and NLRX1 have all been reported in the context of *H*. *pylori* infection with roles ranging from detection of *H*. *pylori* to initiation of inflammation or regulatory responses at the gastrointestinal mucosa [16, 18]. We analyzed the three-phase response clusters for known PRRs in an effort to dissect complex induction of immunity and potential molecular crosstalk specific to macrophages co-cultured with *H*. *pylori*. Statistical comparison between uninfected and infected samples at every time point highlighted significant temporal behavior for each PRR (FDR p-value < 0.05). Every measured PRR transcript, except *Tlr12*, demonstrated differential expression at one or more time points. Up-regulated PRRs conformed to either sustained or late expression patterns; no transient peaks were observed (**Fig 2A**).

Extracellular receptors *Tlr2* and *Tlr6* were significantly co-expressed beginning at 60 minutes and remained relatively stable over time. *Tlr1* was the only other up-regulated cell-surface receptor and was more strongly expressed later during infection. TLR2 is essential for recognizing *H*. *pylori* by dendritic cells and therefore it is not unexpected to find its transcript at significantly high levels during our co-culture. Moreover, TLR2 represents the only known mechanism whereby *H*. *pylori* induces a significant regulatory signature, which is in line with our clustering results above (Fig 1, C4) [19, 20]. Lipoproteins from *H*. *pylori* and other microbes can signal through either a TLR1/2 or TLR2/6 heterodimer however this has not been extensively investigated [21, 22]. Other extracellular PRR mRNAs were repressed (*Tlr4*, *Tlr5)*, unchanged (*Tlr12*), or undetected (*Tlr10*, *Tlr11)* upon bacterial colonization (**Fig 2B**).

With regards to intracellular PRRs, *Nlrp3* and *Nod2* both ranked as highly significant transcripts and were induced early but maintained relatively stable levels over time. *Nod1*, *Nlrc5*, *Ifih1* and *Dhx58*, were induced in a pattern akin to the late wave and begin immediately after the peak of intracellular *H*. *pylori* replication measured by bacterial re-isolation. NOD1 is recognized as a primary contributor of host response to *H*. *pylori* through the detection of intracellular peptidoglycan, a component of the bacterium’s cell wall, and is likely a key detection method used by macrophages to identify infiltrated bacteria in our project [5]. The roles and influence of the other aforementioned intracellular receptors have been well documented in the context of *H*. *pylori* infection and recently reviewed [23]. We expanded our analysis to encompass inflammasome components and found that indeed *Casp-1* and *Casp-11* were both significantly induced at late time points. Notably, Caspase-11 was recently identified as an innate receptor for intracellular LPS and although *H*. *pylori-*derived components do not directly induce Caspase-11 it has not been confirmed whether the live bacterium can be recognized through this novel mode of detection [24]. Additionally, caspase proteins require post-translational modifications to become active and their involvement in immune responses to *H*. *pylori* must be confirmed at the protein level.

The majority of PRRs participate in host immunity by inducing inflammatory responses in immune cells. Indeed the patterns of extracellular and intracellular receptors are in line with intracellular *H*. *pylori* replication and time-specific activation of sustained and late cytokine waves. However, one novel subgroup of NLRs, whose members include NLRC3, NLRP12 and NLRX1, functions to attenuate inflammation by regulating activation of NF-kB and MAVS signaling pathways [25]. Our transcriptomic profiling demonstrates that macrophages treated with *H*. *pylori* have significantly repressed *Nlrc3* and *Nlrx1* transcripts, especially early post-infection; a finding further validated by qRT-PCR along with sustained and late-wave genes (**Fig 2C**). These data are aligned with recent reports demonstrating that *H*. *pylori* infection suppresses *Nlrx1* and *Nlrp12* in a human monocytic cell-line [18]. *Nlrp12* was detected at 360 minutes only in our dataset and it was slightly increased. Suppression or deficiency of regulatory NLRs has been linked to worsened disease due to exacerbated inflammation and cell death however mechanisms underlying the negative-feedback loops that govern this phenomenon are not well understood [26–28].

### Modeling macrophage responses to intracellular replication of *H*. *pylori* reveals possible mechanisms of regulation of innate responses by NLRX1

Due to substantiated evidence that NLRX1 can be suppressed in an *H*. *pylori* dependent manner [18], we hypothesized that NLRX1 represents a central molecular checkpoint involved in the fate of bacterial burden and macrophage responses during infection. To investigate the roles of effector versus regulatory PRRs during *H*. *pylori* infection in macrophages we generated a calibrated ODE-based model representing the pathways implicated in NLRX1-mediated immunity.

#### Network model

**Fig 3:**
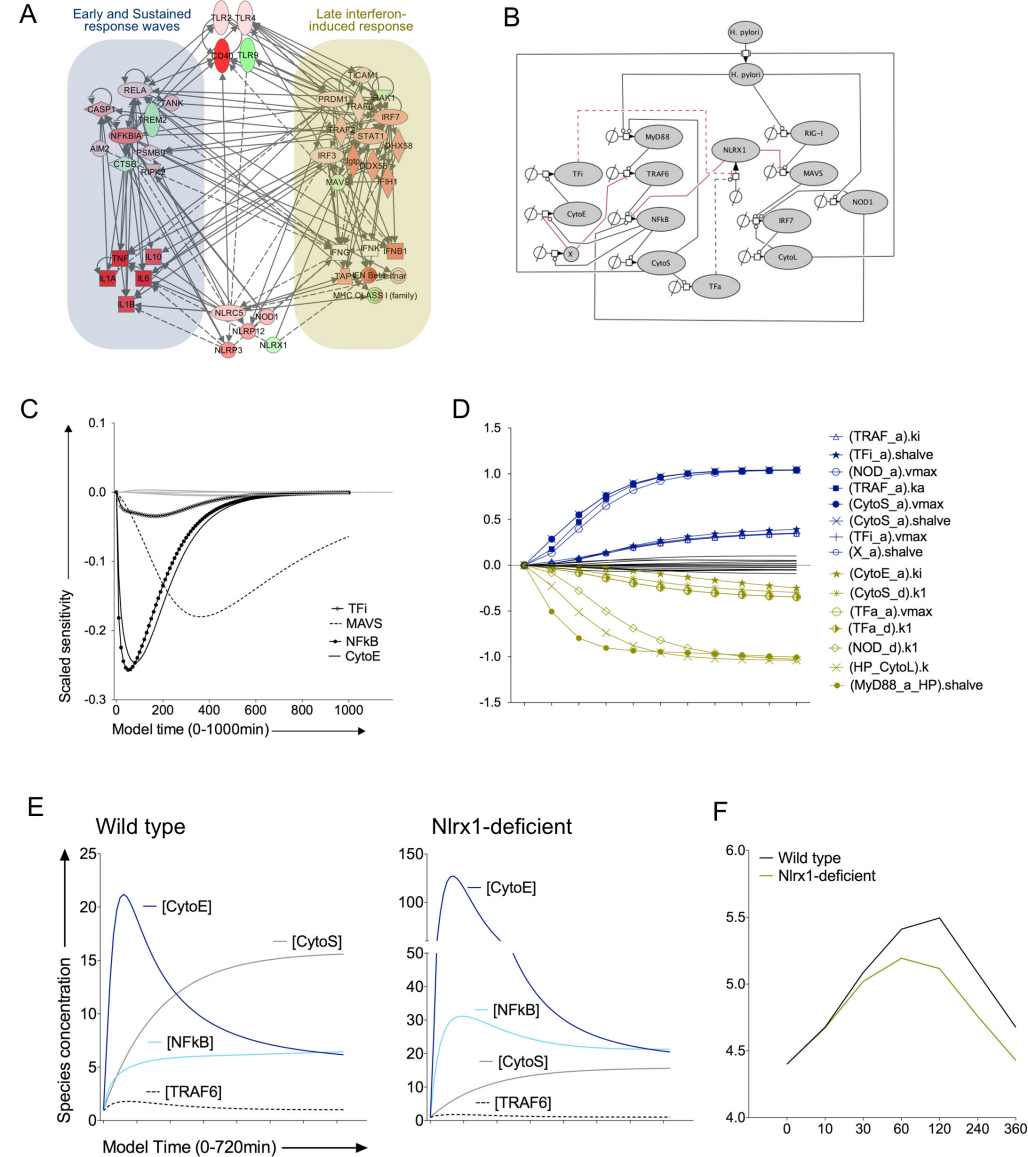
Modeling macrophage immunity to *Helicobacter pylori* infection reveals an essential role for NLRX1 in favoring bacterial burden. Differentially regulated genes from the RNAseq dataset were analyzed for molecular crosstalk using Ingenuity Pathway Analyzer and a preliminary interaction network was created. Red indicates increased expression whereas green indicates reduced expression at time 360 min. Molecules in white are not detected (A). Early, sustained and late wave responses were included. Feedback circuits were added to canonical pathways and a biological system was engineered in Systems Biology Mark-up Language (B). In B: red lines represent negative regulation, positive reactions are black, solid lines are experimentally validated and dashed lines are hypothetical feedback loops. Two sensitivity analyses were performed on the calibrated mathematical model: effects of model species on NLRX1 (C); and effect of NLRX1 on local parameters (D). Time course model simulations for a wild type compared to Nlrx1-deficient system demonstrate differences in NF-kB signaling (E) and *H*. *pyori* burden *in silico* (F).

Despite the highly specific nature of PRRs, our bioinformatics analysis suggests high levels of redundancy, crosstalk and synergism among these sentinel inflammatory receptors; a common pattern that manifests in host response to bacteria [16]. For instance, *H*. *pylori* detection through TLR2 and NOD1 can both result in ample NF-κB activation and inflammatory cytokine production. However, detection by PRRs is dependent upon bacterial location (e.g. TLR2 is extracellular whereas NOD1 is intracellular) resulting in pathway activation at different spatiotemporal scales. Cytokine signaling triggers positive feed-forward loops that further activate the same molecular profile (i.e. TRAF6 phosphorylation occurs due to detection of bacteria (e.g. TLRs) and cytokines (e.g. TNFR)). Additionally, NF-κB signaling is tightly controlled through numerous self-regulated negative feedback mechanisms, including PPARγ and NLRX1 [29]. To generate a comprehensive model structure we first performed network analysis on differentially expressed genes using the Ingenuity Pathway Analysis (IPA) tool [30]. Relationships in top altered canonical pathways were merged to encompass the three temporal gene-expression waves (**Fig 3A**). We integrated IPA canonical pathways with feedback loops and generated a systems biology markup language (SBML)-compliant model that incorporates recognition of *H*. *pylori* through TLR2 (via adaptor molecule MyD88), NOD1 and RIG-I. Pathogen detection initiates second-tier inflammatory cascade branches that activate either NF-kB or IRF3/7 (IRF) and ultimately result in the production of early (CytoE), sustained (CytoS) and late (CytoL) cytokine responses. Negative feedback from NF-kB is represented cumulatively by inhibitory factor X. NLRX1 functions to regulate inflammation by inhibiting MAVS and the activation of NF-kB byTRAF6 (**Fig 3B**).

Causative agents for suppression and resolution of NLRX1 are unknown. Therefore, we engineered our model in an effort to describe such behavior. Based on our analysis of expression patterns described in previous sections, *Nlrx1* decreases when early transient responses are high and increases as sustained and late cytokines rise. Thus we added hypothetical negative and positive feedback loops from undefined transcription factors induced by early and sustained responses, respectively, to NLRX1. These feedback loops represent two key predictions of NLRX1 modulation embedded in our computational model. Out of the 120 early gene set, 17 have transcription factor or nucleic acid binding capabilities and represent potential candidates for negative feedback. In addition to genes extracted from the early cluster above, *Bcor*, *Irf4*, *Myc* and *Rel* fall within this category. Positive feedback could be linked to several genes in the sustained or late waves such as NF-κB molecules, *Stat5* (both in C9), *Stat1* or *Irf7* (C11).

#### Training data and parameter fitting results

In order to study the kinetic behavior of our reaction network, we generated a deterministic mathematical model wherein each species was assigned an ODE (**S1 Fig**). We combined in-house bacterial re-isolation with select data from our transcriptomic profiling results to calibrate the system of equations. Each model species was trained using data measured at six distinct time points for its respective gene transcript. Unknown rate values were estimated using COPASI [31, 32]. Specifically the model was optimized to fit experimental data by minimizing a weighted sum of squared residuals using a Genetic Algorithm. After training, the model accurately simulates the three distinct waves of macrophage immunity to *H*. *pylori*. Fitting results for parameters and model species to experimental training datasets are presented in (**S2 Fig**).

#### Sensitivity analyses

After calibration we assessed the local sensitivities of NLRX1 on transient concentrations of other model species. Our results suggest that NLRX1 plays an inhibitory role in all three host-response waves. Initially NLRX1 will negatively regulate NFκB signaling and early response factors. However NLRX1 will serve as a repressor of MAVS signaling at the onset of late signaling (**Fig 3C**). We calculated a second sensitivity analysis to determine the effects of local model parameters on the temporal behavior of NLRX1. Activation of MyD88 signaling by *H*. *pylori* had the largest negative impact on NLRX1 expression over time. Although high quantities of *H*. *pylori* are inhibitory in the beginning, accelerated bacterial clearance due to late cytokines causes the second largest negative impact on NLRX1. This suggests a complex pattern of interaction in that the presence of *H*. *pylori* may both promote and inhibit NLRX1 expression depending on spatiotemporal detection and prevailing immune responses (**Fig 3D**). In contrast, model parameters required for sustained cytokine production were positively influential for NLRX1. The importance of NOD1 in positively regulating NLRX1 was an unexpected finding and further supports the existence of crosstalk between effector and regulatory NLRs. Interestingly, parameters controlling NFκB, RIG-I and MAVS kinetics had very little influence on NLRX1.

#### *In silico* NLRX1-deficient system

We next generated an *in silico* knockout system to predict the effects of NLRX1-deficiency on macrophage immunity to intracellular *H*. *pylori*. To create the knockout system, initial and transient concentrations of NLRX1 were fixed at a constant value of 0. The most dramatic differences in the NLRX1-null system are depicted by elevated NF-κB activity. Interestingly, increased NF-κB levels caused a strong response skewed toward up-regulated early cytokines while sustained cytokines remained unchanged (**Fig 3E**). Although the loss of NLRX1 did exaggerate MAVS concentrations, downstream IRF and late cytokines were unaffected. Lastly, increased NF-κB and early wave molecules in the NLRX1-/- model were directly associated with reduced *H*. *pylori* burden in our model. Specifically, bacterial replication peaks around 60 minutes in the NLRX1-deficient system compared to 120 minutes in the WT system with the most dramatic differences occurring 120 minutes onward (**Fig 3F**).

To summarize, our modeling and simulation studies generated four computational hypotheses surrounding the immunomodulatory mechanisms of macrophage-NLRX1 during *H*. *pylori* infection. First, *H*. *pylori* rapidly induces an early wave response that is largely responsible for NLRX1 suppression in infected macrophages. Second, sustained inflammatory mediators are required for the induction of NLRX1 expression during infection. Third, the loss of NLRX1 results in a sharp increase of NF-κB and early cytokines but little changes are observed in sustained or late cytokines. Lastly, NLRX1-deficiency results in lower levels of intracellular *H*. *pylori* when compared to wild type simulations.

### Validation studies demonstrate that NLRX1-deficiency reduces intracellular *H*. *pylori* loads

**Fig 4:**
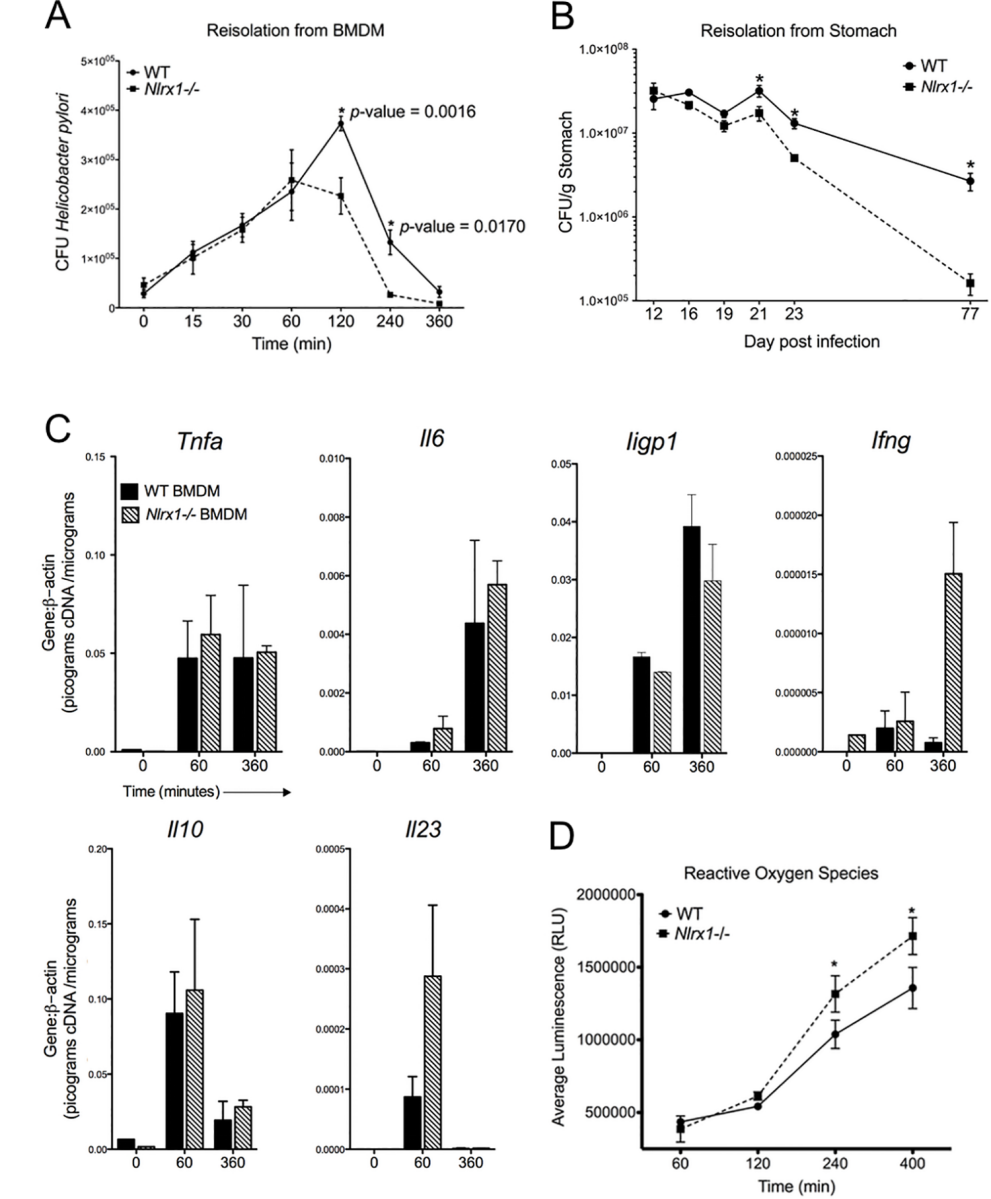
Experimental validation studies confirm role for NLRX1 in *Helicobacter pylori* burden and host immunity. Bone marrow derived macrophages were obtained from wild type and *Nlrx1-/-* mice then challenged with *H*. *pylori* using the gentamycin protection assay. Cells were collected incrementally from 0 to 360 minutes post co-culture and assayed for intracellular *H*. *pylori* replication via re-isolation (n = 3) (A), gene expression via quantitative real-time RT-PCR (C), or production of reactive oxygen species (n = 3) (D). Stomachs from *H*. *pylori* infected mice were collected from 12 to 77 days post infection and bacterial burden was determined via re-isolation (n = 7) (B). Asterisk indicates *P*-value < 0.05 based on ANOVA.

Using computational modeling predictions as a governing framework, we performed a series of validation experiments to confirm the immunomodulatory role of NLRX1 in macrophage immunity to *H*. *pylori*. BMDM were generated from wild type and *Nlrx1-/-* mice, subjected to the gentamycin protection *H*. *pylori* co-culture, and then assayed for bacterial re-isolation or quantitative gene expression. We validated the computational hypothesis that NLRX1 deficiency alone is sufficient for reducing bacterial burdens when compared to wild type cells (**Fig 4A**). Moreover, bacterial levels indeed peak earlier, around 60 minutes, in *Nlrx1-/-* versus wild type BMDM mirroring our *in silico* predictions. *Nlrx1-/-* mice experienced significantly lower gastric bacterial loads when compared to their wild-type counterparts beginning 21 days post infection. Differences in *H*. *pylori* colonization between genotypes became more accentuated over time and diverged over ten-fold by 77 days post-infection (**Fig 4B**). Due to the lack of conditional NLRX1 knockout animals, our murine studies do not confirm cell-specific roles of *H*. *pylori* infected macrophages *in vivo* however this presents an opportunity for future validation. Next, gene expression was assayed using real-time RT-PCR at 0, 60, and 360 minutes during co-culture to assess early (*Il10*, *Il23*), sustained (*Tnf*, *Il6*) and late (*Iigp1*) response transcripts. NLRX1-deficient BMDM behaved similar to wild-type samples and up-regulated appropriate genes in a time-dependent manner. Almost no differences were observed in genes measured in sustained and late waves emulating observations presented by *in silico* experiments. Differences were observed in *Il23* at 60 minutes validating model predictions, however differential regulation of *Il10*, another early cytokine, was not observed. Since IL-10 and IL-23 are differentially regulated by bacterial stimulation, these data do not necessarily negate one another [33]. Since *H*. *pylori-*infected epithelial cells and monocytes often produce IFN-γ as a means of antimicrobial host immunity we measured its transcript even though it is not included in our model [34]. Interestingly, *Ifng* mRNA levels increase dramatically at 360 minutes in macrophages lacking NLRX1 presenting another possible mechanism whereby NLRX1-deficenct cells reduce pathogen loads (**Fig 4C**). Finally, we measured reactive oxygen species (ROS) production due to its involvement in NLRX1-mediated immunity and *H*. *pylori* resistance to host mechanisms [34]. Similar to *Ifng*, BMDM lacking NLRX1 produced significantly higher amounts of ROS when compared to wild type macrophages during infection with intracellular *H*. *pylori* (**Fig 4D**).

These studies have provided novel mechanistic insights toward characterizing the cell-specific roles of NLRX1 in macrophages. Collectively our data validates the computational prediction that NLRX1-deficient macrophages infected with *H*. *pylori* exert dysregulated host responses resulting in a microenvironment that favors bacterial clearance. We also established that reduced *H*. *pylori* colonization due to the lack of NLRX1 is paralleled *in vivo*. Our findings support computational hypotheses that point away from classically associated NF-κB and type-I-interferon signaling and suggest a novel role for type-III-interferons (IFN-γ) and ROS production in NLRX1-mediated regulation of *H*. *pylori*. Moreover, they help form a picture for a role of NLRX1 in mediating host tolerance to *H*. *pylori*.

## Discussion

*H*. *pylori* has co-evolved with its human host for over 60,000 years [35, 36], diverged with ancient human migrations [37], and infects half of the world’s population. Individual responses toward the bacterium vary tremendously with respect to strain, host, genetic, and socioeconomic factors resulting in differences ranging from asymptomatic superficial gastritis to invasive adenocarcinoma [2, 38]. Approximately 10–15% of infected individuals develop peptic ulcers, gastric carcinoma or mucosa-associated lymphoid tissue lymphoma as a result of improperly controlled gastritis at the stomach mucosa [39]. In contrast, a larger cohort of carriers remains asymptomatic or develops beneficial immunity due to *H*. *pylori* colonization. Specifically, emerging data reveal *H*. *pylori*-dependent protection against esophageal and cardial pathologies [40–44], childhood asthma and allergies [45–48], inflammatory bowel disease [49] and obesity in developed countries [50, 51] demonstrating symbiotic properties of this bacterium. Cell populations and molecular mechanisms that mediate host tolerance by actively shaping immune responses to minimize detrimental effects of co-existence with high levels of *H*. *pylori* are likely critical for orchestrating pathogenic versus commensal roles of this bacterium [52].

This study aimed to broadly elucidate immunomodulatory properties of intracellular *H*. *pylori* on macrophages. Similarly we sought to determine prevailing immunity employed by macrophages to either favor or counterpoise intracellular invasion. To do so, we generated the first time-series global gene expression dataset for *H*. *pylori* infected macrophages and identified three novel response modules: early transient, sustained and late waves. Additionally, we describe how temporal behavior of differentially expressed transcripts is induced by redundant, synergistic and antagonistic detection mechanisms of extracellular and intracellular PRRs suggesting a level of spatiotemporal dependency. From these studies we confirm that macrophage-*H*. *pylori* co-culture suppresses NLRX1 and present novel evidence that NLRC3, a member in the same subset of regulatory NOD-like receptors, is also significantly repressed. By developing a calibrated computational model of host responses to *H*. *pylori* in macrophages we predict, then experimentally validate, that NLRX1 is a key determinant for maintaining elevated *H*. *pylori* loads at the cellular and organismal levels. Importantly, we propose a novel mechanism underlying *H*. *pylori-*induced reduction of NLRX1 governed by a negative-feedback loop from the early-induced transient response element. Taken together, this work provides a novel and comprehensive framework for dissecting perturbations in innate immunity caused by *H*. *pylori* and could help reveal novel broad-based mechanisms implicated in the maintenance of host tolerance to a variety of organisms.

Considerable evidence has surfaced demonstrating that MNP populations are highly influential in governing prevailing mucosal immunity, pathogen loads and gastric pathology during *H*. *pylori* infection. Cells from a hematopoietic origin have been shown to provide protection against T-cell mediated gastritis in *H*. *pylori* infected mice [53]. Enrichment of certain phenotypes such as CD103+CD11b-IL10+ dendritic cells during infection bestows protective systemic immunity in *H*. *pylori-*colonized hosts. Our team recently identified a novel subset of CD11b+F4/80hiCD64+CX3CR1+ MNPs that expand upon infection, secrete high levels of IL-10 and are required for maintaining elevated levels of *H*. *pylori* in the stomach mucosa early during infection. Modulating macrophage phenotypes by genetic ablation of transcriptional machinery, such as hypoxia-inducible factors [54] or peroxisome proliferator-activated receptor gamma, results in loss of protection characterized by substantially lower *H*. *pylori* colonies at the expense of mucosal damage. Molecular mechanisms prompting myeloid cells to provide protective immunity and restrict inflammatory signals in the gastric mucosa are largely unexplored. In terms of overall cellular response, our data suggests that effector responses from BMDM co-cultured with *H*. *pylori* occur in patterns similar to dendritic cells [16], especially TLR2 induced genes and NOD1-mediated immunity. One principal advantage of our dataset is that we capture the entire early transient signaling wave and thus we describe the regulatory element of macrophage immunity in more detail than previous studies [16, 55]. Beyond IL-10 we have described five new regulatory pathways and validating their physiological roles in host tolerance will be valuable next steps for understanding potential mechanisms governing protective or deleterious effects of pathogens and members of the human microbiota.

Chronic persistence of *H*. *pylori* is generally attributed to the microbe’s numerous immune-evasion tactics [37]. CagA+ *H*. *pylori* strains are rapidly ingested by macrophages but survive in the intracellular compartment due to their ability to resist phagocytic killing and form viable colonies in megasomes [56]. Additional evidence demonstrates that *H*. *pylori* can induce autophagic vesicles that can serve as a replication reservoir for the bacterium [57–59]. More recently, emphasis has been on anti-inflammatory responses induced by *H*. *pylori* that promote tolerance and are incidentally related to the protective immunomodulatory properties of the bacterium [60]. In this study we took a host-centric view and investigated dynamic macrophage-dependent mechanisms involved in detecting and regulating intracellular survival of *H*. *pylori*. We analyzed the activity of PRRs and found that all measured PRR transcripts were significantly altered at one or more time points with the exception of *Tlr12*. Presently, NOD1 and TLR2 are the main receptors associated with detection of *H*. *pylori*, and indeed we observed upregulation of these molecules in our dataset. Importantly, we report for the first time that *H*. *pylori* stimulation of macrophages significantly enhances mRNA levels of *Caspase11*, a non-canonical caspase protein recognized as a receptor for cytosolic lipopolysaccharide (LPS) [24, 61]. *H*. *pylori* has low efficacy for many PRRs partially as a result of the molecular evolution of its surface molecules [3, 62]. *H*. *pylori* LPS has achieved structural mimicry akin to human blood antigens by resembling polysaccharides found in mammalian hosts and thus is nearly undetectable by the LPS-receptor TLR4 [63]. Hence, detection of *H*. *pylori* LPS through an alternative pathway would yield a significant contribution in understanding how the host can recognize intracellular *H*. *pylori* and should be validated at the protein level. Interestingly, many effector PRRs were repressed over time, including TLR5. To note, TLR5 mRNA and protein levels are shown to increase when measured in other *H*. *pylori* co-cultures whereas our data demonstrates significant down-regulation of the molecule. Since we are utilizing an intracellular infection method, it is possible that this co-culture system does not yield adequate extracellular bacteria levels for proper TLR5 activation. Additionally, these differences may be attributed to cell-specificity or timing; previous reports employ 24-hour co-cultures [64]. Modulating expression and cellular distribution of PRRs following host-microbe interaction is a fundamental tactic used by immune cells to alter sensitivity to bacteria and reduce the inflammatory impact of stimulation. Classically this property is affiliated with the intestinal environment where adjusting expression and bio-geographical distribution of PRRs by epithelial cells and MNP is directly involved in the balance between homeostasis and development of autoimmune diseases such as inflammatory bowel disease [65]. Based on our findings it is tempting to speculate that macrophages modify PRR abundance during *H*. *pylori* infection as a mechanism to circumvent susceptibility to overt inflammatory disease.

One key finding of our study was reduced expression of regulatory NLRs, NLRC3 and NLRX1, following perturbation of innate immune responses by *H*. *pylori*. Suppression of regulatory NLRs is associated with worsened pathology during inflammatory diseases but mechanisms of negative regulation have not been described [26–28]. We and others have demonstrated that NLRX1 can be suppressed in an *H*. *pylori* dependent manner [18], however limited mechanistic insight exists to explain this behavior or its contribution to immune responses and bacterial burden. To further investigate the roles of NLRX1 during *H*. *pylori* we engineered a calibrated ODE-based computational model that encompassed the three-wave innate immune response identified by our clustering analysis. Using *in silico* knockout simulations we predicted and validated the novel hypothesis that, despite being suppressed during peak microbial loads, NLRX1 is required for maintaining high levels of *H*. *pylori* in both macrophages and the murine stomach. Analytical results on our computational model propose a novel scenario for immunomodulation in which host responses rapidly induced following *H*. *pylori* colonization of macrophages regulate NLRX1 suppression; a prediction is being investigated intensively in ongoing validation studies.

Another key validation experiment confirmed model predictions that sustained (i.e. *Tnf* and *Il6*) and late (i.e. *Iigp1*) effector molecules were largely unchanged in NLRX1-deficient BMDM compared to wild type during the gentamycin protection assay. Our studies do show significant differences in the induction of *Ifng* and ROS in NLRX1-null macrophages occurring through an undefined mechanism. Importantly, one model-driven hypothesis is that loss of NLRX1 would result in higher levels of early cytokines. We indeed observed higher levels of the cytokine transcript for *Il23* in *Nlrx1-/-* BMDM. Il-23 has been studied with several infectious disease models and is largely involved in regulating robust innate immunity required for containing bacterial burden [66]. On the other hand, early *Il10* response measurements were very similar between genotypes. IL-23 and IL-10 signal through different mechanisms and are differentially regulated in mononuclear phagocytes infected with various pathogens, thus these pathways may be working independently [33] and future studies could investigate the contrast in signaling with respect to NLRX1. It remains unclear whether lower bacterial loads represent more efficient killing of *H*. *pylori* by macrophages, reduced replication rates due to altered intracellular singling (i.e. autophagy), or both. NLRX1 is believed to antagonize NF-κB activity, modulate reactive oxygen species (ROS), promote autophagy and interrupt interferon signaling [67] via direct interaction with intermediate transduction proteins, TRAF6 and MAVS. These findings, however, remain controversial and vary among murine models of infectious and immune-mediated diseases. For example, NLRX1 has been identified as a regulator of mitochondrial antiviral immunity through interference with RIG-I-MAVS and TRAF6-NF-kB signaling to attenuate inflammation during Sendai virus and influenza infections and LPS induced shock [68–71]. In contrast, *Nlrx1-/-* mice have reduced interferon responses during influenza independent of MAVS interactions [72, 73]. Similarly, the debate that NLRX1 potentiates ROS production [74, 75] or not [76] is ongoing. Since NLRX1 serves as a cytosolic receptor for host and microbe components, it is likely that NLRX1 acts in a pathogen-specific manner, potentially contributing to conflicting results. Our results support the notion that NLRX1 negatively influences ROS production. Additionally we present IFN-γ as a unique identifier for altered immunity resulting from loss of NLRX1.

A potential limitation of our findings could result from the slight misfit of early cytokines in our model. Experimental data suggests that the early response wave reaches values closer to baseline and more rapidly than our model. One possible explanation to rationalize this result is the lack of an unforeseen feedback loop that negatively regulates early responses independent of NF-κB. Some early genes, such as *Ddit4*, are involved in autophagy and regulated by lipid and glucose metabolism suggesting roles for both classical NF-κB and alternative enzymatic signaling [77]. Future model extensions could incorporate metabolic and autophagy modules to enhance fitting of initial transient signaling components and provide clarity for induction of this novel regulatory element. Likewise, we did not predict the result of IFN-γ since type III interferon signaling was not included in our present model; this branch of immunity could be added in future renditions. Additionally, it is possible that the inverse behavior observed between early response elements and NLRX1 is coincidental rather than causal. Given over 100 genes in the early response element, it is not feasible to experimentally test each molecule’s ability to control transcriptional activity of NLRX1. Future model versions with factorial simulations or sparse sensitivity analyses designs will help narrow more likely candidates for validation. Lastly, the use of only one *H*. *pylori* strain (SS1) in our experimental validation is a limitation in characterizing role of *H*. *pylori* virulence factors on the expression of host PRR signaling. Strain SS1 is positive for cagA and cagE virulence factors but vacA inactive [78]. Interestingly, the only other study on NLRX1 behavior during *H*. *pylori* co-culture shows comparable results using an *H*. *pylori* strain negative for cagA and cagE yet positive for vacA [18]. This suggests that NLRX1 regulation may be more dependent on host response elements rather than bacterial influence however this speculation will need to be confirmed in future studies.

In conclusion, we combined transcriptomic analyses, mathematical modeling and experimental validation of key computational predictions to characterize novel macrophage-dependent mechanisms involved in detection and response to intracellular *H*. *pylori*. The regulatory response element that we describe involves genes that have not yet been connected with disease outcomes during *H*. *pylori* infection thus presenting an exciting new avenue for potential investigation. Through analytical studies of a calibrated mathematical model, we determine that the dynamic behavior of one molecule, NLRX1, is tightly regulated and essential for *H*. *pylori* colonization. Concisely, persistently high levels of microbial colonies can only occur when NLRX1 is involved in a feedback loop that suppresses sustained and late immune waves. Finally, our integrated computational and experimental validation studies also shed light on an undescribed role for IFN-γ in NLRX1 immunity as well as a unique scenario for host-mediated suppression of NLRX1. Broadly, our model of host response to *H*. *pylori* infection represents an invaluable tool to study the mucosal immune system as a massively interacting information processing system. Moreover, our findings present new data that could assist in the development of host tolerance-based precision medicine strategies that do not rely on anti-microbial treatment.

## Materials and Methods

### Ethics Statement

All experimental procedures were approved by the Institutional Animal Care and Use Committee of Virginia Tech and met or exceeded the requirements of the Public Health Service, National Institutes of Health and the Animal Welfare Act. Animals were housed two to fiver per cage on a ventilated rack with a standard 12:12 light/dark cycle and were under strict monitoring throughout the duration of the project. After infection, animals were monitored daily for signs of disease and all efforts were made to minimize unnecessary pain and distress. If animals succumbed to excessive morbidity (>20% weight loss), animals were scarified prior to the designated experimental endpoint. No animals experienced significant morbidity or mortality in our experiments. Mice were euthanized by carbon dioxide narcosis followed by secondary cervical dislocation. Animal experimentation was performed under IACUC protocol 12–174 VBI.

### Isolation and culture of bone marrow derived macrophages (BMDM)

Bone marrow was isolated from hind legs obtained from wild type and *Nlrx1-/-* mice in sterile conditions as previously described [79]. Briefly, muscle was removed and bones were soaked in 70% ethanol for 1 minute. Bones (femur and tibia) were cut proximal to each joint and flushed with 10mL cold sterile complete macrophage medium (RPMI 1640, 10% FBS, 2.5% Hepes, 1% Sodium pyruvate, 1% Pen/Strep, 1% L-Glutamine) using a 25-G needle inserted into the bone marrow cavity. Cells were filtered through a 100 μm strainer and centrifuged for 8min at 1,200 rpm at 4°C. Red blood cells were eliminated by osmotic lysis. Single cell suspensions of bone marrow progenitors were plated in untreated petri dishes with media supplemented with 25ng/mL murine macrophage colony stimulating factor (M-CSF, Peprotech). Cells were fed on day 3 with fresh media containing M-CSF. On day 6 of culture, nonadherent cells were removed whereas adherent cells were collected by scraping.

### *H*. *pylori* culture

*H*. *pylori* growth plates were prepared with Difco Columbia blood agar base (BD Biosciences) supplemented with 7% horse blood (Lampire) and *Helicobacter pylori* selective supplement (containing 10 mg/liter vancomycin, 5 mg/liter trimethoprim, 5 mg/liter amphotericin, and 5 mg/liter polymyxin from Oxoid) at 37°C under microaerophilic conditions as previously described [7].

### Gentamicin protection assay

BMDM cell suspensions were resuspended in antibiotic-free cRPMI and seeded at a density of 250,000 in 12-well plates for bacterial reisolation or 2 x 10^6^ in 6-well plates for RNA isolation and left overnight to adhere at 37°C, 95% humidity and 5% CO_2_. The following day, cells were co-cultured with *H*. *pylori* strain SS1 at an MOI 10 and synchronized by quick spin to ensure immediate bacteria-cell contact. After a 15-minute incubation, extracellular bacteria was killed and removed by washing cells with PBS/5%FBS containing 100ng/ml Gentamicin. Complete macrophage media supplemented with 100ng/mL Gentamicin was added to cells and the co-culture proceeded for the indicated times. Time-point 0 represents the initial collection beginning after Gentamicin washes. At desired time points, cells were washed with 1xPBS and collected by scraping for downstream assays. For re-isolation of *H*. *pylori* from cells, BMDM were collected in 100μl of sterile Brucella broth and sonicated for 5 sec to release intracellular bacteria; serial dilutions were plated under microaerophilic conditions described above.

### Global transcriptomic gene expression analysis by RNAseq

Total RNA was isolated from WT BMDM collected at time-points 0, 60, 120, 240, 360 and 720 minutes post-coculture with *H*. *pylori* strain SS1. Each time point contained triplicates for *H*. *pylori* treated and non-infected controls. RNA was submitted for whole transcriptome gene expression assessment using Illumina Hiseq (Virginia Bioinformatics Institute Core Lab Facilities). Fastq files containing 100bp-long pair-end reads were assessed and poor quality reads (>40% of bases with PHRED score <10; percentage of N greater than 5%; and polyA reads) were filtered out. Remaining high quality reads were mapped to RefSeq (mm10 from http://genome.ucsc.edu/) using Bowtie [80] (version: 1.0.0) with parameters set to ‘-l 25-I 1-X 1000-a-m 200’. Gene expression levels were calculated using an expectation-maximum algorithm, RSEM [81]. FPKM [82] (fragments per kilobase per million sequenced reads) values were used as expression level measurements. The present RNAseq dataset has been submitted to NCBI's GEO database (Accession Number GSE67270).

### Clustering

Hierarchical based clustering was used to analyze the time series RNAseq data. Expression patterns of significant genes (FDR p-value < 0.05) were used to group genes into different clusters. The clustering was performed in R (version 3.1.3) using the *hclust* method with Ward's minimum variance method and Manhattan distance metric. The clustering and Gene Ontology analysis on the sub-clusters were performed iteratively to group genes in ideal sets; 12 sets were found to be ideal for a well separated functional group from this dataset. Genes of low significance and minor kinetic changes grouped together during clustering and were not explored in depth in our analysis. Therefore, clustering was considered an additional layer for filtering following statistical analysis.

### Computational Modeling

Mathematical equations and parameter values for our computational model are presented in **S1 Fig** Unknown parameter values were estimated using COPASI [31, 32]. Specifically the model was optimized to fit experimental data by minimizing a weighted sum of squared residuals using a Genetic Algorithm. The experimental training dataset used for model was calibrating was transcriptomic levels obtained by RNAseq at six distinct time points for wild type BMDM stimulated with *H*. *pylori* as outlined above. Two or three replicates were provided per transcript per time point for training. Due to high distribution in FPKM readouts, replicates are representative of the fold change of *H*. *pylori* infected cells normalized by time-matched untreated cells. Thus concentrations of model species over time is indicative of fold change (SS1 treated/untreated). Model species-transcript pairing was as follows: NLRX1-*Nlrx1;* NOD1-*Nod1;* RIGI-*Ddx58*; MAVS-*Mavs*; IRF7-*Ifr7*; CytoL-*Iigp1* and–*Ifi47;* MyD88-*Myd88;* TRAF6-*Traf6*; NFkB-*Nfkbia;* CytoE-*Il10* and–*Il23;* CytoL-*Il1b* and–*Il6*. Data and fitting are presented in **S2 Fig.** Sensitivity analyses were also calculated in COPASI as previously described [83]. In this case, sensitivity analysis of NLRX1 on local parameters was performed with respect to reaction rates in the system whereas sensitivity analysis on transient concentrations of NLRX1 was performed with respect to kinetic fluxes of local model species.

### Validation of gene expression by qRT-PCR

RNA was isolated from BMDM using the RNeasy Minikit following manufacturer’s instructions (Qiagen). mRNA concentrations were quantified by optical density at 260 nm with a Nanodrop spectrophotometer (Invitrogen). One microgram of RNA per sample was used to synthesize cDNA using the iSCRIPT cDNA Synthesis kit (Bio-Rad). Quantitative reverse transcription (RT)-PCR was performed (CFX96; Bio-Rad) to assess absolute expression of genes (primers listed in **S1 Table**). Standard curves were created by diluting primer-specific PCR amplicons of known concentrations (5 to 5×10^−6^ pg per reaction volume).

### Detection of secreted reactive oxygen species

ROS was measured by monitoring bioluminescent activity of hydrogen peroxide species in culture via the ROS-Glo H_2_O_2_ Assay (Promega). Briefly, the enzymatic substrate provided by the kit was added to cells in culture during the gentamycin protection co-culture following manufacturer’s instructions for direct cell-based detection. The assay was performed in a 96-well plate with a density of 10,000 cells in 100uL media. Cells were protected from light and incubated for up to 6 hours with luminescent ROS-Glo reagents.

### Murine model of *H*. *pylori* infection

Age and sex matched 8–12 week-old wild type and NLRX1-/- mice from a C57B/6 background were challenged with *Helicobacter pylori* strain SS1 by orogastric gavage after a 6-hour fast. *H*. *pylori* inoculum was prepared fresh in sterile 1X PBS and was administered to mice on days 0 and 2 of the study (500 μl, 5x10^7^ CFU/mouse). Between weeks 2 and 11 post-infection mice were euthanized and stomach was excised for bacterial re-isolation. Gastric tissue was thoroughly rinsed in sterile 1X PBS, weighted, then homogenized in Brucella broth using a BioVortexer and pestle (Thomas Scientific). Serial dilutions were plated onto Difco Columbia agar base plates as described above.

### Statistics

For statistical analysis on our RNAseq dataset, all genes with a median expression level in all samples greater than 0 were included in a 2-way (treatment and time) ANOVA analysis. Normal quantile transformation (qqnorm from R [84]) was used to normalize the FPKM to fit the normality assumption of ANOVA (tested with Kolmogorov-Smirnov test). Two-way ANOVA analysis was performed in R [84]. FDR [85] and Bonferroni adjustments were used to identify differentially expressed genes.

## Supporting Information

S1 Fig Ordinary differential equation based math model and respective parameter values for NLRX1-mediated macrophage immunity. Dynamic behavior for each species in the biological system of NLRX1-mediated immunity in macrophages (Fig 3B) was obtained using ordinary differential equations. Parameter values were estimated in COPASI.
(DOCX)

S2 Fig Model fitting to experimental training dataset. Simulations for each model species (solid black line) compared to respective training data (dots). All training data, except for *Helicobacter pylori*, was extracted from our time course RNAseq dataset for wild type bone marrow derived macrophages co-cultured with *H*. *pylori*. Training data for *H*. *pylori* represent two separate projects and each dot represents the average of three replicates. A Genetic Algorithm in COPASI was used to fit data and calculate parameter values.
(DOCX)

S1 Table Nucleotide sequences and accession numbers used to design primers for quantitative real-time RT-PCR. (DOCX)
