# Supplementary material for: Modeling the Regulatory Mechanisms by Which NLRX1 Modulates Innate Immune Responses to Helicobacter pylori Infection
Source: PLoS One. 2015 Sep 14;10(9):e0137839. doi: 10.1371/journal.pone.0137839 (PMC4569576; doi:10.1371/journal.pone.0137839)
Supplement: S1 Fig — Dynamic behavior for each species in the biological system of NLRX1-mediated immunity in macrophages (Fig 3B) was obtained using ordinary differential equations. Parameter values were estimated in COPASI. (DOCX) [file pone.0137839.s001.docx]

**
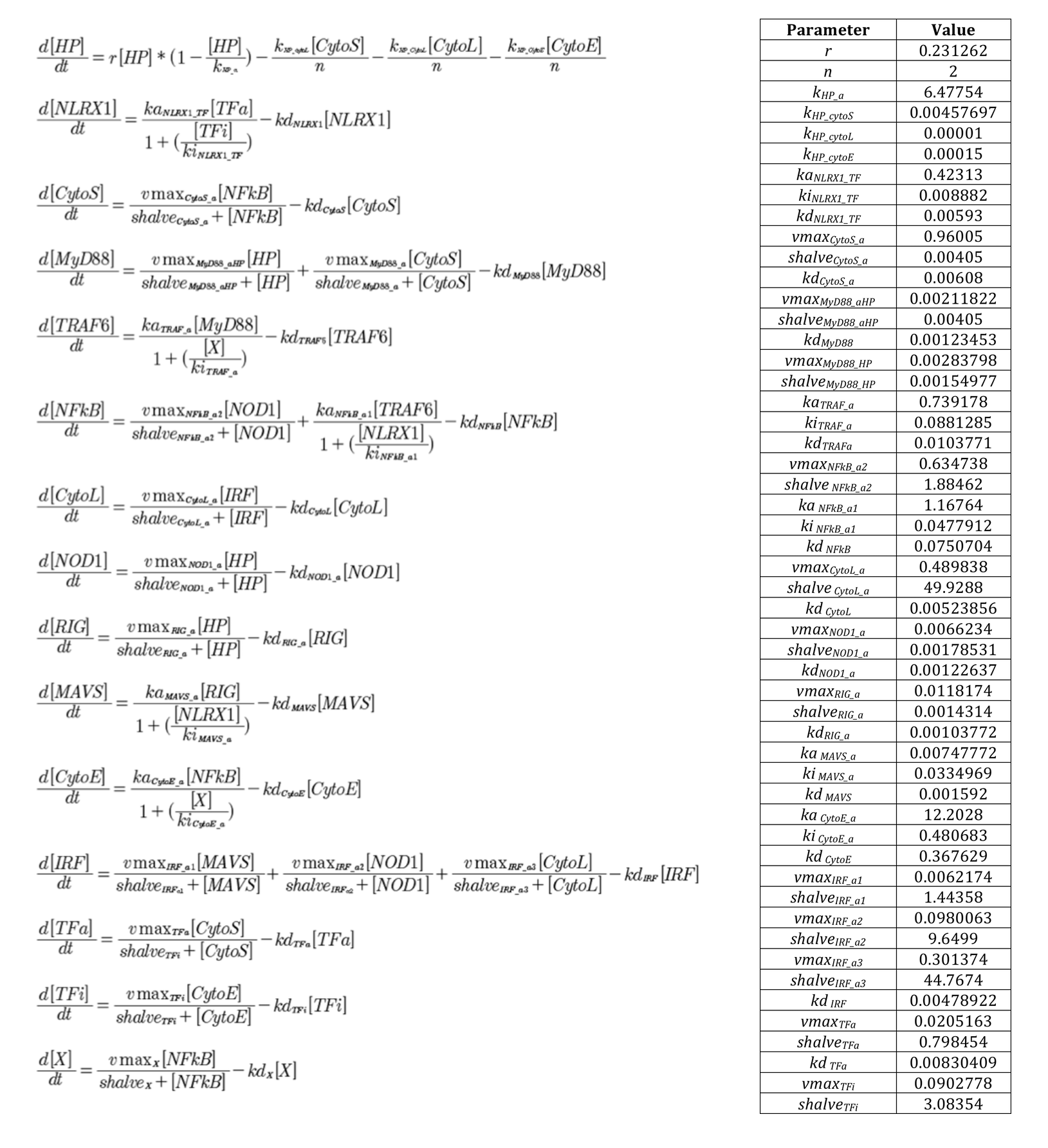
**

**S1 Fig. Ordinary differential equation based math model and respective parameter values for NLRX1-mediated macrophage immunity.** Dynamic behavior for each species in the biological system of NLRX1-mediated immunity in macrophages (Figure 3B) was obtained using ordinary differential equations. Parameter values were estimated in COPASI.
