# Supplementary material for: Modeling the Regulatory Mechanisms by Which NLRX1 Modulates Innate Immune Responses to Helicobacter pylori Infection
Source: PLoS One. 2015 Sep 14;10(9):e0137839. doi: 10.1371/journal.pone.0137839 (PMC4569576; doi:10.1371/journal.pone.0137839)
Supplement: S2 Fig — Simulations for each model species (solid black line) compared to respective training data (dots). All training data, except for Helicobacter pylori, was extracted from our time course RNAseq dataset for wild type bone marrow derived macrophages co-cultured with H. pylori. Training data for H. pylori represent two separate projects and each dot represents the average of three replicates. A Genetic Algorithm in COPASI was used to fit data and calculate parameter values. (DOCX) [file pone.0137839.s002.docx]

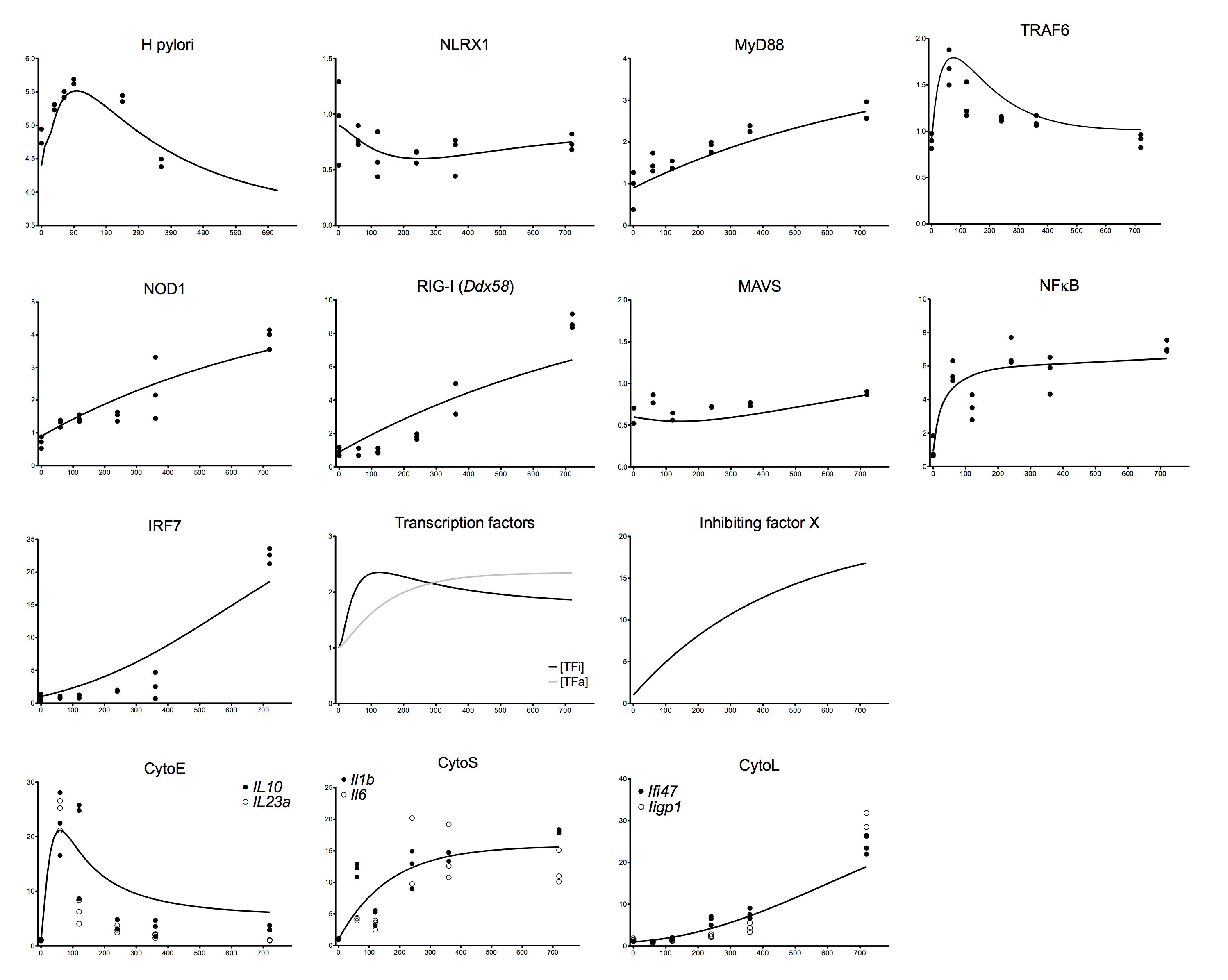


**S2 Fig. Model fitting to experimental training dataset.** Simulations for each model species (solid black line) compared to respective training data (dots). All training data, except for *Helicoabcter pylori,* was extracted from our time course RNAseq dataset for wild type bone marrow derived macrophages co-cultured with *H. pylori*. Training data for *H. pylori* represent two separate projects and each dot represents the average of three replicates. A Genetic Algorithm in COPASI was used to fit data and calculate parameter values.
