# Supplementary material for: Modeling the Regulatory Mechanisms by Which NLRX1 Modulates Innate Immune Responses to Helicobacter pylori Infection
Source: PLoS One. 2015 Sep 14;10(9):e0137839. doi: 10.1371/journal.pone.0137839 (PMC4569576; doi:10.1371/journal.pone.0137839)
Supplement: S1 Table — (DOCX) [file pone.0137839.s003.docx]

| **S1 Table. Nucleotide sequences and accession numbers used to design primers for quantitative real-time RT-PCR.** | | |
| --- | --- | --- |
| Accession Number | Primer | Sequence |
| NM_010548 | IL-10 F  IL-10 R | 5’ GGG TTG CCA AGC CTT ATC GGA AAT-3’  5’ TCT TCA GCT TCT CAC CCA GGG AAT-3’ |
| NM_031252 | IL-23p19 F  IL-23p19 R | 5’ CCT TAG TGC CAA CAG CTT AA-3’  5’ AGA TGT CTG GGC TGA TAG ATT-3’ |
| NM_031168 | IL-6 F  IL-6 R | 5’ CCT GCC TAA TCG ACA GAC TG-3’  5’CTG AAG GAC TCT GGC TTT GT-3’ |
| NM_013693 | TNFα F  TNFα R | 5’ ACT GCC AGA AGA GGC ACT CC-3’  5’ CGA TCA CCC CGA AGT TCA-3’ |
| NM_021792 | ligp1 F  ligp1 R | 5’ ACC TGC AAA TTC TGT CTC A-3’  5’ TGT ATG TCC ATG TAC CAT ATA AAC-3’ |
| NM_010927 | NOS2 F  NOS2 R | 5’ GCT TTG TGC GAA GTG TCA GT-3’  5’ CTC CTT TGA GCC CTT TGT G-3’ |
| NM_178420 | NLRX1 F  NLRX1 R | 5’ TCT CAA CCT CAA TTT CCA-3’  5’ AGC AGA TTG ACA ATG ATC-3’ |
| NM_008337 | IFNγ F  IFNγ R | 5’ TAA TTA GCC AAG ACT GTG AT-3’  5’ ACT GAG AGT CCT AGA AAG CAT AA-3’ |
| X03672 | β-actin F  β-actin R | 5’CCC AGG CAT TGC TGA CAG G-3’  5’ TGG AAG GTG GAC AGT GAG GC-3’ |
